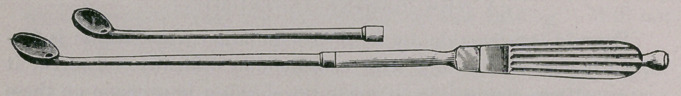# Department of Canine, Feline, and Avian Medicine and Surgery

**Published:** 1902-04

**Authors:** Cecil French

**Affiliations:** D.V.S. (McGill and Munich), Washington, D. C.


### DEPARTMENT OF CANINE, FELINE, AND AVIAN MEDICINE AND SURGERY.

By Cecil French, D.V.S. (McGill and Munich),

WASHINGTON, D. C.

### A NEW CANINE INSTRUMENT.

Rectal obstruction by fecal accumulation (coprostasis) is frequently removable only with considerable difficulty. Indeed, it is sometimes impossible to make the slightest impression on these hardened masses by the simple method of attacking them with instruments through the anal orifice, and it then becomes necessary to open the abdominal cavity, and either force the matter on by manipulation or deliver it by enterotomy.

Now, while the operation of cœliotomy on a healthy dog is ordinarily remarkably free from any ill-effect, it is a different matter when the animal is septicæmic. Rinne (*Ueber den Eiterungsprocess und seine Metastasen*, Berlin, 1889, p. 61), in a series of experiments on dogs, observed that injection of sterilized putrid fluids together with staphylococci into the peritoneal cavity was succeeded by suppuration of all open wounds, which otherwise healed kindly, thereby demonstrating that pyogenic cocci could be derived from the circulation. An animal which has suffered any length of time from fecal obstruction is in a condition of copræmia, or, in other words, its blood is charged with the products of intestinal putrefaction together with the micro-organisms causing the same. It is in a condition very similar to that produced artificially by Rinne; and it is small wonder that wounds, particularly of the peritoneal cavity, under these circumstances show a pronounced tendency to heal unkindly, and even terminate unfavorably. Hence, any measures involving interference with the integrity of that part of the organism should only be employed as a last expedient. Experience has taught me to persevere by simple means when attempting to relieve fecal impaction.

The instruments commonly employed in this condition are the rectal scoop or curette and the rectal forceps, supplemented by the rectal syringe.

As I have already intimated, one may sometimes work for hours with the instruments mentioned without making any pronounced headway, owing to the hardness of the concrement, its distance from the anal orifice, or the swollen state of the rectal mucosa; but if one alternately uses the scoop and directs a stream of water against the mass it may be more easily disintegrated and softened. This fact made it evident to me that an instrument was needed which would combine both features of scooping and irrigating capacity.

Accordingly, I had Haussmann & Dunn manufacture a scoop, the stem of which is hollowed to permit of a steady flow of water on the point of the mass being attacked. The extremity of the handle receives the rubber tubing of the syringe, and the water escapes at the base of the bowl. The stem is made in two sizes, either of which is screwed into the handle, as desired. The water should be injected by means of a bulb syringe, as a fountain syringe lacks the requisite force.

But there is another feature about this instrument that makes it doubly valuable. It has been a matter of observation that if a considerable volume of water can be conducted beyond the obstructing mass the bowel will often become sufficiently distended and stimulated to produce its evacuation without any further assistance. The instrument, being of good length, permits of its employment for this purpose.

---

THERAPEUTIC USE OF LECITHIN. Lecithin influences the general nutrition of the organism, and especially that of the nervous system. Lancereaux and Paulesco have employed the ovolecithin, 50 centigrammes daily, to counteract the progressive depression from pancreatic diabetes. The emaciation ceased, the weight increased, and the general state ameliorated. Analogous effects were seen in other diverse vices of nutrition.

Desgrez and Zaki administered to the guinea-pig and the dog lecithin prepared from hens' eggs. The former were divided into three lots and fed on bran-bread and cabbage. The first lot were "checks;" the second received hypodermically, every two days, 0.06 grain of lecithin; the third received the same dose by the mouth, in pill form. After forty-three days the increase in weight was 43 per cent. for the first, 60 per cent. for the second, and 75 per cent. for the third lot; urea and other nitrogenous excretions were increased. The results with the dogs were analogous.—*Ann. de Méd. Vét.*

## Figures and Tables

**Figure f1:**